# Diagnostic Value of Neutrophil-to-Lymphocyte Ratio, Lymphocyte-to-Monocyte Ratio, and Platelet-to-Lymphocyte Ratio in the Diagnosis of Erythema Nodosum Leprosum: A Retrospective Study

**DOI:** 10.3390/tropicalmed7030039

**Published:** 2022-03-02

**Authors:** Natalia Tanojo, Damayanti, Budi Utomo, Evy Ervianti, Dwi Murtiastutik, Cita Rosita Sigit Prakoeswa, Muhammad Yulianto Listiawan

**Affiliations:** 1 Department of Dermatology and Venereology, Faculty of Medicine, Universitas Airlangga/Dr. Soetomo General Academic Hospital, Jl. Mayjen Prof. Dr. Moestopo No. 6–8, Surabaya 60286, Indonesia; liatanojo@gmail.com (N.T.); damayanti@fk.unair.ac.id (D.); evy_if@yahoo.co.id (E.E.); dwimurtiastutik@yahoo.co.id (D.M.); cita-rosita@fk.unair.ac.id (C.R.S.P.); 2 Department of Public Health and Preventive Medicine, Faculty of Medicine, Universitas Airlangga, Jl. Mayjen Prof. Dr. Moestopo No. 47, Surabaya 60132, Indonesia; budiutomo@fk.unair.ac.id

**Keywords:** neutrophil-to-lymphocyte ratio, lymphocyte-to-monocyte ratio, platelet-to-lymphocyte ratio, erythema nodosum leprosum, leprosy, tropical disease, infectious disease, neglected disease

## Abstract

Erythema nodosum leprosum (ENL) is an acute immune complex-mediated condition of the dermis, subcutaneous tissue, and other tissues seen in patients with multibacillary (MB) leprosy, causing severe impairment to patients’ quality of life. To date, there is no standard diagnostic criteria for ENL. We aimed to study the diagnostic value and accuracy of Neutrophil-to-Lymphocyte ratio (NLR), Lymphocyte-to-Monocyte ratio (LMR), and Platelet-to-Lymphocyte ratio (PLR) in diagnosing ENL. This is an analytic retrospective study with a cross-sectional design that describes the distribution and clinical characteristics of all newly diagnosed MB patients of Dr. Soetomo General Hospital Surabaya in the years 2018–2020. NLR, LMR, and PLR were calculated for all patients, and a receiver operating characteristic curve (ROC) was generated to identify the cut-off points. Among a total of 182 patients with MB leprosy, 22 cases (12.09%) were reported with ENL. WBC, neutrophils, monocytes, and thrombocytes showed a positive correlation with the incidence of ENL, but not lymphocytes. The NLR cut-off point for the diagnosis of ENL was 4.99 (sensitivity 86.4%, specificity 82.5%, accuracy 82.97), while that of PLR was 237.46 (sensitivity 63.6%, specificity 73.1%, accuracy 71.98%). LMR had poor sensitivity and specificity levels of 50% and 28.7%, with cut-off point of 2.28 and accuracy of 31.32%. These results suggest that NLR and PLR could be potential biomarkers for the diagnosis of ENL.

## 1. Introduction

Leprosy is one of 20 neglected tropical diseases caused by *Mycobacterium leprae*, with more than 200,000 new cases reported every year from more than 120 countries [1,2]. The clinical manifestations of leprosy vary depending on the host immune response. In 1981, the WHO introduced a simple disease classification based on the number of skin lesions and the finding of acid-fast bacilli (AFB) to aid the prescription of multidrug regimens. Paucibacillary (PB) leprosy is associated with five or less skin lesions and negative AFB findings, while multibacillary (MB) patients present with more than five skin lesions and positive AFB findings [2]. The majority of leprosy cases in Indonesia, which has the third highest number of global leprosy cases, are MB leprosy (84%) [3,4].

Erythema nodosum leprosum (ENL) is an acute inflammatory complication of leprosy that exclusively occurs in MB leprosy [5,6]. It presents as a sudden onset of generalized painful erythematous nodules with or without symptoms of systemic inflammation that may occur before, during, or after multi-drug therapy (MDT) [6,7,8]. The clinical criteria proposed by B. Naafs et al. for leprosy diagnosis are considered sufficient in daily clinical practice, although histopathological findings such as findings such as increased vascularity with dilated capillaries in the dermis with neutrophil infiltration may aid in disease confirmation [9]. ENL has frequent recurrence, with the possibility to persist for years [10]. Leprosy patients with ENL are prone to nerve damage and potential physical disabilities which severely impact their quality of life, the social and economic burden, and patients’ mortality [7,8,10,11]. The diagnosis of ENL often relies on the medical expertise of doctors and their ability to recognize the disease’s clinical manifestations [10]. The histopathology of ENL resembles that of MB leprosy without ENL, except for the possible presence of neutrophil infiltration, microabscesses, or vasculitis [9]. The increase of acute-phase proteins, such as C-reactive protein, gamma globulin, α1-antitrypsin, and α1-acid glycoprotein, may help the diagnosis, although related tests are often expensive and not available [10].

ENL is described as a neutrophilic immune-complex-mediated condition with a complex interaction of multiple immune system elements, including monocytes, lymphocytes, and platelets [5,12]. Neutrophil infiltration is the histological hallmark of ENL [12]. A recent study indicated that the number of peripheral neutrophils, especially of low-density neutrophils (LDNs), is increased especially in severe ENL [6]. Monocytes are rarely studied in ENL, although the expression of tumor necrosis factor (TNF)-α is higher in patients with MB leprosy with ENL than in those without ENL [12]. Platelet or thrombocyte helps to trap pathogens during cellular immunity by forming microthrombi [9]. The involvement of lymphocytes in ENL is often limited to increased T-helper lymphocytes and decreased T-cytotoxic lymphocytes, with an increase of the T-helper-to-T-cytotoxic lymphocyte ratio in comparison to the control group. B-lymphocytes are not associated with the formation of ENL [12].

Recently, the Neutrophil-to-Lymphocyte ratio (NLR), Lymphocyte-to-Monocyte ratio (LMR), and Platelet-to-Lymphocyte ratio (PLR) have been used in different conditions to reflect the host inflammatory response, hence enabling medical providers to confirm disease diagnosis, predict the prognosis, and monitor treatment outcome. A study by Gomes et al. [10] indicated that NLR was 78% accurate in diagnosing leprosy reactions.

This study aimed to study the diagnostic value of NLR, LMR, and PLR as biomarkers in diagnosing ENL in endemic leprosy.

## 2. Materials and Methods

This was a cross-sectional retrospective study of data obtained from medical records of patients who presented, between January 2018 and December 2020, at the Leprosy Division of dr. Soetomo General Academic Hospital, Surabaya, Indonesia. Eligible patients were males and females aged 18 years and above, with a confirmed diagnosis of MB leprosy from skin lesions and AFB analysis, who were subjected to a complete blood test on the same day of diagnosis. Patients with HIV, secondary syphilis, hypertension, and diabetes mellitus were excluded. Patients with a history of steroid and other immunomodulators use in the last two weeks were excluded, due to the possibility of host immunity alteration. Ethical clearance was obtained from the Ethical Committee of Dr. Soetomo General Academic Hospital, Surabaya, with reference number 0459/LOE/301.4.2/V/2021.

Physicians experienced in the diagnosis and treatment of leprosy determined the diagnosis of leprosy based on WHO criteria, which include the finding of hypopigmented or erythematous skin lesion(s) with impairment or loss of sensation, peripheral nerves thickening or sensory impairment, or positive acid-fast bacilli smear. Patients were further classified into MB leprosy when presented with six or more lesions with or without positive bacterial index. Patients who did not fulfil the criteria were classified as PB leprosy and excluded from this study. ENL was clinically determined by the findings of a sudden eruption of tender papules, nodules, or plaques and three of the following symptoms: mild fever, tender enlarged nerves, arthritis, lymphadenitis, increased loss of sensation or muscle power, epididymo-orchitis, edema of the extremities or face, positive Ryrie or Ellis test, and iridocyclitis or episcleritis. The same physicians obtained demographic data including comorbidities, gender, age, and the onset of ENL through anamnesis and performed tests necessary to determine physical disabilities, which were graded using the WHO 2-point scale. Bodyweight and height were measured for body mass index (BMI) calculation to determine the nutritional status based on the cutoff for Asian and Asian Americans. The bacterial and morphological indexes were determined from skin smears obtained from three different sites. The number of white blood cells and differentials, including neutrophils, monocytes, and lymphocytes, as well as thrombocyte counts, were recorded and used to calculate NLR, LMR, and PLR by dividing the absolute count of neutrophils by that of lymphocytes, the absolute count of lymphocytes by that of monocytes, and the absolute count of thrombocytes by that of lymphocytes, respectively.

A descriptive analysis was performed for all study variables and reported as median (minimum–maximum) for non-parametric data and mean (±standard deviation) for parametric data. Two-times-two Pearson’s chi-square analyses were done on gender, BI, and MI data, while the non-parametric Kruskal–Wallis test was used to determine the relationship between other variables and ENL. The correlation between blood counts and incidence of ENL was analyzed by using Spearman’s rho correlation test. A receiver operator characteristic (ROC) curve was constructed to evaluate the sensitivity and specificity of NLR, LMR, and PLR.

## 3. Results

A total of 280 new patients visited the Leprosy Division in January 2018–December 2020, including 6 subclinical leprosy, 6 neural leprosy, 13 PB leprosy, and 255 MB leprosy cases. Approximately 98 subjects were excluded from the study due to age, diagnosis of non-MB, or absence of laboratory results. Out of the 182 cases included in this study, only 22 cases (12.09%) were diagnosed with ENL. The 22 ENL patients were free from any comorbidity, while the non-ENL patients were reported to have HIV (1.65%), diabetes mellitus (1.09%), Cushing syndrome (0.55%), and secondary syphilis (0.55%).

Table 1 showed that most ENL patients were men (72.72%) aged 18–40 years (68.18%) who had never received multi-drug therapy (45.45%) or with a history of complete MDT treatment (31.82%). Grade 2 disability (G2D) was found in 9.09% of newly diagnosed ENL patients. Nutritional status was determined through BMI measurement and indicated that most ENL cases had normal BMI (59.09%). Most ENL cases had a BI of less than 3 (77.27%) and an MI of less than 5 (90.9%). The onset of ENL treatment was the only variable that was significantly different between the ENL and the non-ENL groups (*p* = 0.001).

Table 2 indicates that the median WBC of ENL subjects (14.820 (4,160–30,330); *p* = 0.001) was almost twice higher than that of subjects without ENL (7.405 (4.380–21.980)). Neutrophil count in the ENL group (12.355 (3,520–26,840); *p* = 0.001) was almost three times higher than that in the non-ENL group (4,840 (2,010–19,540)). Monocyte count was just slightly higher in ENL cases (775 (210–1,910); *p* = 0.051), while lymphocyte count was lower (1,526.82 (±655.57), *p* = 0.159), but none of these two values showed a statistically significant differences between the two groups. The medians of NLR, LMR, and PLR in ENL cases were 8.19 (2.9–21.46), 2.28 (0.75–4.94), and 283.97 (126.48–1,267.65), respectively. NLR and PLR revealed a positive correlation with the incidence of ENL with coefficients of 0.45 and 0.26, respectively, while LMR showed a negative correlation, with a coefficient of −0.205.

**Figure 1:**
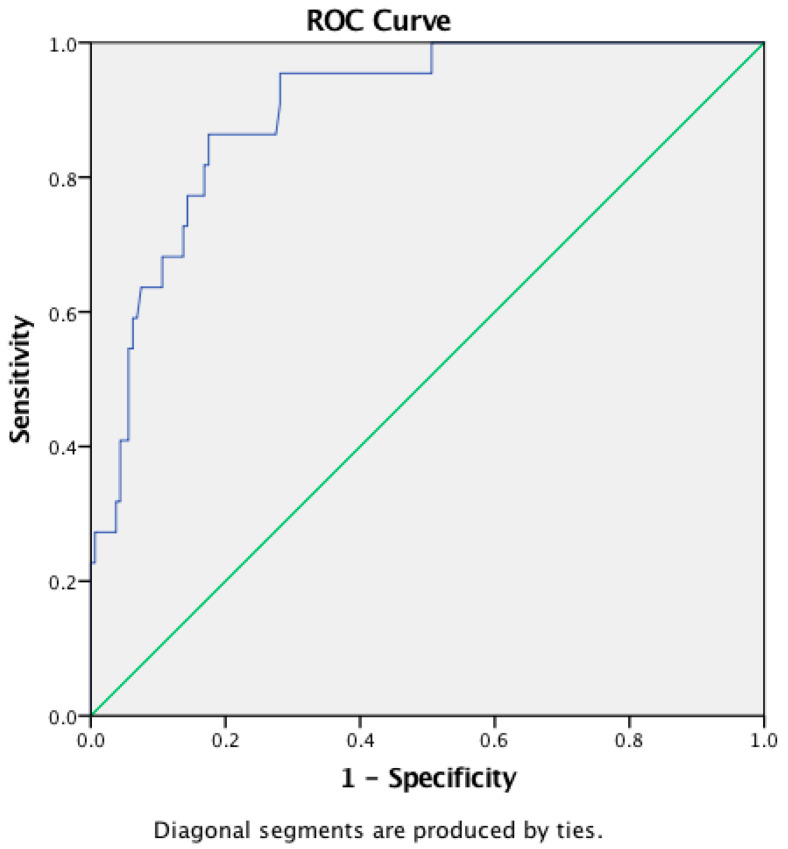
ROC curve of NLR.

**Figure 2:**
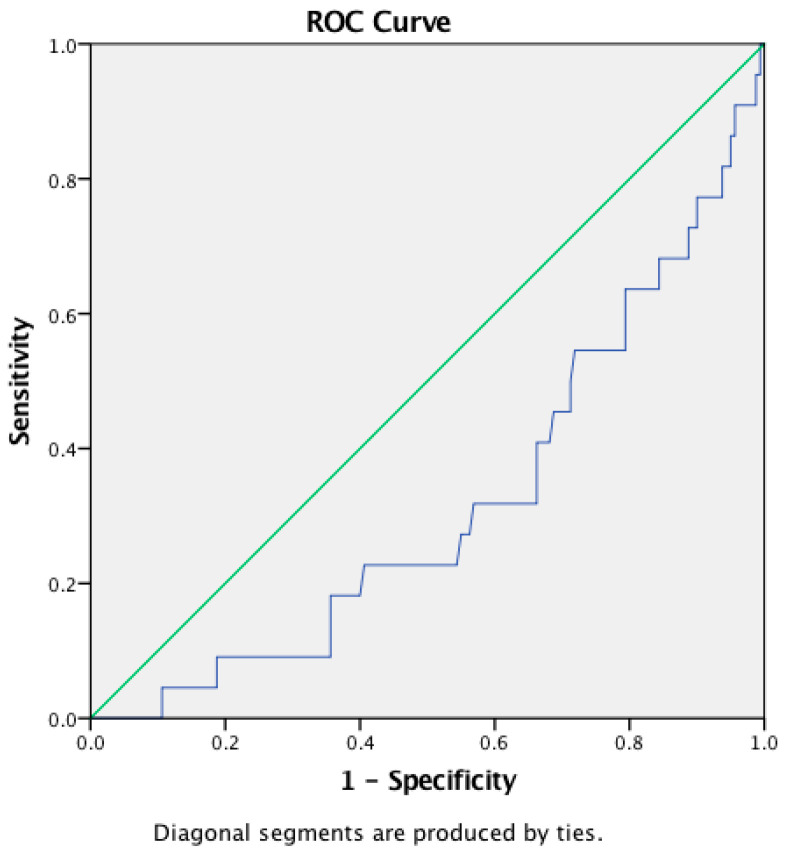
ROC curve of LMR.

**Figure 3:**
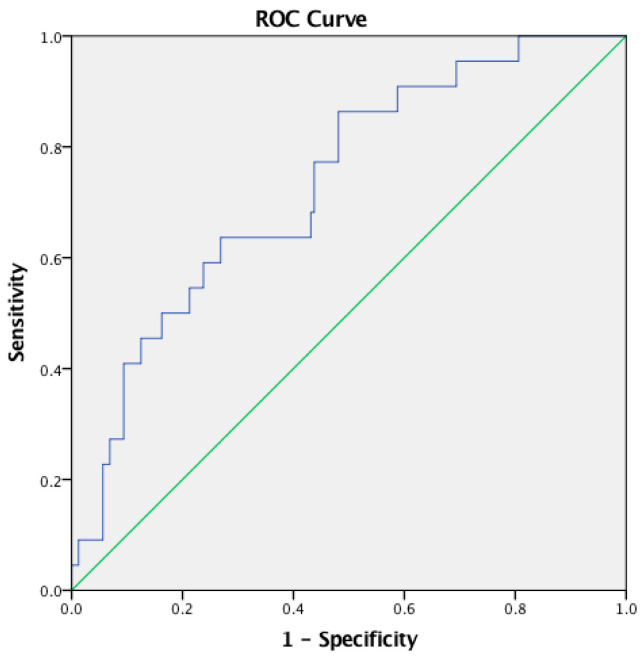
ROC curve of PLR.

The AUCs of NLR, LMR, and PLR were 0.899, 0.318, and 0.732 for the diagnosis of ENL, respectively. The NLR cutoff point for the diagnosis of ENL was 4.99 (sensitivity 86.4%, specificity 82.5%, accuracy 82.97%) (Figure 1), while for LMR, it was 2.28 (sensitivity 50%, specificity 28.7%, accuracy 31.32%) (Figure 2), and for PLR, 237.46 (sensitivity 63.6%, specificity 73.1%, accuracy 71.98%) (Figure 3).

## 4. Discussion

This study describes the distribution and clinical characteristics of ENL, showing a relatively low incidence corresponding to 12.09% of all new MB patients. A previous study by Fransisca C., et al. [13] in the same hospital in 2015–2017 reported a higher ENL incidence of 33% in all MB cases. This difference may be related to the fact that in this study, we eliminated a group of patients, who could have been suffering from ENL but did not perform the necessary laboratory analysis to complete the study. Comorbidities, such as HIV, secondary syphilis, hypertension, and diabetes mellitus may complicate the disease manifestations and hinder treatment [14]. None of the ENL-positive subjects in this study had comorbidities or a history of medications that might affect the results.

The demographic data showed that ENL predominantly occurred in normal BMI male aged 18–40 years with or without a history of MDT treatment. The slit-skin smear analysis indicated that the majority of ENL subjects had a BI of less than 3 (77.27%) and an MI of less than 5 (90.9%). None of these predominant characteristics displayed a significant difference with respect to the non-ENL group, except for the treatment onset. MDT is an effective treatment against *M. leprae* that has successfully suppressed the incidence of leprosy since its introduction in 1981 [15]. Ironically, MDT used for leprosy treatment, which leads to bacterial fragmentation, is hypothesized to elicit the formation of antigen–antibody complexes in ENL [12]. The cause–effect relationship between MDT treatment and ENL explains the higher incidence of ENL found in patients on MDT therapy or after being released from MDT, in comparison to newly treated patients [12,13]. The incidence of ENL during MDT can be at least twice higher than that at the time of initial diagnosis [11].

Macrophages, which are tissue monocytes, are the main resident cells activated by *M. leprae* infection [6,16]. Earlier studies indicated that ENL patients experienced a greater release of TNF-α from monocytes upon contact with *M. leprae* [12,17]. However, the number of circulating monocytes did not differ between groups with and without leprosy reactions [12,18]. Monocytes are unlikely to actively participate in the pathogenesis of ENL even though the immune complex reaction in ENL involves intricate interactions between antigens released from foamy macrophages and antibodies generated by lymphocytes [9,18].

Neutrophils, the most abundant type of WBC, provide protection through phagocytosis, the release of antimicrobial peptides, and the generation of neutrophils extracellular traps [12]. Histological studies showed an intense perivascular infiltrate of neutrophils throughout the dermis and subcutis, especially within 72 h of ENL onset [11,12]. The circulating neutrophils appeared to contain *M. leprae* bacilli, albeit in the absence of systemic inflammation [16]. Neutrophilic degranulation was observed in ENL, with a subsequent high production of low-density neutrophils [6]. The released granules acted as mediators of innate and adaptive immunity that inflicted collateral tissue damage in ENL [16].

Lymphocytes are involved in both cellular and humoral immune responses. T-lymphocytes actively eliminate bacterial, viral, and parasitic infections through an adaptive immune response. The antigen specificity of T-cells is based on the major histocompatibility complex molecules presented by antigen-presenting cells, such as skin dendritic cells, macrophages, and B-cells [12]. B-lymphocyte produce the antibodies that form immune complexes leading to the Arthus phenomenon in the pathogenesis of ENL [10]. However, the active involvement of lymphocytes in different aspects of immune reactions makes lymphocyte count unreliable in differentiating ENL from non-ENL [10]. 

Platelets function in the blood coagulation system and fibrinolysis and play a major role in immunothrombosis by promoting microvessel thrombosis that allows capturing pathogens thus limiting pathogen dissemination [19]. Elevated platelet count was observed in ENL patients, especially in the early phase of the disease [19,20]. One of the important mediators of thrombocytosis, interleukin-6, is also elevated during the initial phase of ENL [21].

The involvement of WBCs, including neutrophils, monocytes, and lymphocytes, as well as platelets throughout the pathogenesis of ENL may lead to an increase in cell counts during laboratory analysis. However, in this study, only WBC, neutrophils, and platelets showed a significant difference in their number in patients during ENL episodes in comparison to MB patients without ENL formation. NLR was determined by dividing the absolute neutrophil count by the absolute lymphocyte count. The use of this ratio may help to better evaluate the immune status of the host than the use of the neutrophil or lymphocyte count alone [10]. Nevertheless, NLR is not a specific biomarker, and its value may be affected by hormonal changes, hypovolemic change, the use of oral steroids, hematological diseases, and HIV [22]. Our study described a non-parametric distribution of NLR, with a median of 8.19 (2.9–21.46) in ENL patients with no comorbidity and no history of steroid use, and a median of 2.93 (0.86–12.85) in non-ENL patients. Further analysis showed that the NLR value had a positive relationship with the incidence of ENL (ρ 0.450; *p* < 0.05). The diagnostic value of NLR was found to have a cut-off value of 4.99, with 86.4% sensitivity, 82.5% specificity, and 82.97% accuracy. A previous study by Gomes et al. [10] used a lower cut-off point of 2.95 to diagnose ENL, with a sensitivity of 81% and a specificity of 74%.

The LMR was calculated by dividing the absolute lymphocyte count by the absolute monocyte count. LMR has been used in a variety of tumor and malignancy-related conditions and has been shown up to help establish a prognosis [23]. This study found a median of 3 (0.69–8.67) for LMR, with a negative correlation with the incidence of ENL. The ROC curve of LMR indicated a cut-off point of 2.28, with only 50% sensitivity and 28.7% specificity. Despite the correlation, LMR had very poor sensitivity and specificity in the diagnosis of ENL.

The PLR was calculated by dividing the platelet count by the absolute lymphocyte count. PLR has been used as an inflammatory and prognostic marker associated with various types of cancer [24]. This study found a median PLR in ENL patients of 186.59 (72.96–1,267.65). An increase of PLR had a positive relationship with the incidence of ENL (*p* < 0.05), with a median of 283.97 (126.48–1,267.65). The ROC curve helped to find that a cut-off point of 237.46 displayed 63.6% sensitivity, 73.1% specificity, and 71.98% accuracy in diagnosing ENL.

The diagnosis of ENL often relies on the medical expertise of the examining physicians and their ability to recognize the disease clinical manifestations, while non-expert medical providers have to rely on histopathology findings and the assessment of the levels of acute phase proteins; however, these tests may not be widely available in rural areas of endemic countries, such as Indonesia [9]. This study indicates that NLR and PLR had 82.95% and 71.98% accuracy in diagnosing the occurrence of ENL in MB patients. These biomarkers can be evaluated by performing a simple differential count that is readily feasible and relatively inexpensive. The results of this paper may help medical providers especially in endemic areas of leprosy in the diagnosis of ENL.

This study has some limitations. The diagnosis of ENL in this study was made on the basis of clinical observations only, as a reference test for the diagnosis of ENL does not exist. Histopathological results may help to better confirm the diagnosis of ENL and to solidify the key findings of this study. ENL is more likely to occur during MDT, yet this research was only limited to patients’ initial visits. Therefore, misdiagnosis or under-diagnosis of ENL might have occurred. This is the first study proposing NLR, LMR, and PLR as diagnostic biomarkers of ENL. The relatively high specificity and sensitivity of NLR and PLR, which are simple and low-cost tools, may support clinicians in the diagnosis of ENL. These findings may help improve disease control, thus preventing possible complications and disabilities. Additional studies are needed to confirm the results of this study.

## Figures and Tables

**Table 1:** Clinical characteristics of ENL in new MB leprosy patients.

Characteristics			ENL		
		*n* (%)	Absent	Present	*p*-Value
Gender	Males	131 (71.97)	115 (71.87)	16 (72.72)	0.986 ^a^
	Females	51 (28.02)	45 (28.12)	6 (27.27)	
Age	18–40	104 (57.14)	89 (55.62)	15 (68.18)	0.245 ^b^
	41–59	63 (34.61)	56 (35)	7 (31.81)	
	≥60	15 (8.24)	15 (9.375)	0 (0)	
Treatment	New	146 (80.21)	136 (85)	10 (45.45)	0.001 ^b,c^
Onset	On MDT	5 (2.74)	3 (1.87)	2 (9.09)	
	RFT/RFC	9 (4.94)	2 (1.25)	7 (31.81)	
	Dropout	22 (12.08)	19 (11.87)	3 (13.63)	
Disability	0	125 (68.68)	112 (70)	13 (59.09)	0.236 ^b^
	1	37 (20.32)	30 (18.75)	7 (31.81)	
	2	20 (10.98)	18 (11.25)	2 (9.09)	
Nutritional	Underweight	32 (17.58)	28 (17.5)	4 (18.18)	0.545 ^b^
Status	Normal	89 (48.90)	76 (47.5)	13 (59.09)	
	Overweight	49 (26.92)	44 (27.5)	5 (22.72)	
	Obese	12 (6.59)	12 (7.5)	0 (0)	
Bacterial	<3	146 (80.21)	129 (80.62)	17 (77.27)	0.083 ^a^
Index	≥3	36 (19.78)	31 (19.37)	5 (22.72)	
Morphological	<5	160 (87.91)	140 (87.5)	20 (90.90)	0.919 ^a^
Index	≥5	22 (12.08)	20 (12.5)	2 (9.09)	

ENL: Erythema nodosum leprosum; ^a^ Pearson’s chi square; ^b^ Kruskal–Wallis test; ^c^ Significant *p* value < 0.05.

**Table 2:** Blood count of new MB leprosy cases.

Blood Count		ENL		Correlation	*p*-Value
		Present	Absent	Coefficient	
WBC ^a^	7,720 (4,160–30,330)	14,820 (4,160–30,330)	7,405 (4,380–21,980)	0.438 ^c^	0.001
Neutrophil ^a^	5,110 (2,010–26,840)	12,355 (3,520–26,840)	4,840 (2,010–19,540)	0.461 ^c^	0.001
Lymphocyte ^b^	1,711.97 (±629.04)	1,526.82 (±655.57)	1,737.43 (±623.13)	−0.105 ^d^	0.159
Monocyte ^a^	590 (200–1,910)	775 (210–1,910)	570 (200–1,450)	0.145 ^c^	0.051
Thrombocyte ^a^	308,000 (116,000–909,000)	375,000 (229,000–909,000)	302,000 (116,000–721,000)	0.263 ^c^	0.001
NLR ^a^	3.19 (0.86–21.46)	8.19 (2.9–21.46)	2.93 (0.86–12.85)	0.450 ^c^	0.001
LMR ^a^	3 (0.69–8.67)	2.28 (0.75–4.94)	3.13 (0.69–8.67)	−0.205 ^c^	0.005
PLR ^a^	186.59 (72.96–1,267.65)	283.97 (126.48–1,267.65)	173.41 (72.96–893.33)	0.262 ^c^	0.001

ENL: erythema nodosum leprosum, WBC: white blood cells, NLR: neutrophil/lymphocyte ratio, LMR: lymphocyte/monocyte ratio, PLR: platelet/lymphocyte ratio; ^a^ Non-parametric distribution; median (minimum–maximum); ^b^ Parametric distribution; mean (±standard deviation); ^c^ Spearman’s rho; ^d^ Pearson correlation test.
